# BUILDING RECOGNITION ACROSS CAMPUS AND TOWN FOR USC’S AGE-FRIENDLY UNIVERSITY INITIATIVE

**DOI:** 10.1093/geroni/igz038.1253

**Published:** 2019-11-08

**Authors:** Paul Nash, Paul Nash, Caroline Cicero

**Affiliations:** 1 University of Southern California, LA, California, United States; 2 University of Southern California, Leonard Davis School of Gerontology, Los Angeles, California, United States

## Abstract

Details of efforts to highlight existing age-related programming on campus, create a new intergenerational experiential learning program, build a social media presence, and include ‘age’ in the university’s diversity efforts will be discussed. Best practices in community engagement will be emphasized including successes with retired employees, alumni, activities programming, and local age-friendly efforts. Discussion includes the benefits of developing a multidisciplinary working group to ensure the Initiative is a university wide effort and the challenges of working in a large, multi-campus research university located in multilayered bureaucratic local jurisdictions. Future goals and collaboration with AFU Global Network partners and aspirations for addressing societal ageism will be addressed.

